# Editorial: Promising photosensitive agents for photodynamic therapy

**DOI:** 10.3389/fphar.2026.1890329

**Published:** 2026-06-26

**Authors:** Bing Yang, Santosh Chokkakula, Ge Hong

**Affiliations:** 1 Department of Medical and Nursing Science, International College, Krirk University, Bangkok, Thailand; 2 Department of Cell Biology, College of Basic Medical Sciences, Tianjin Medical University, Tianjin, China; 3 Department of Microbiology, Chungbuk National University College of Medicine and Medical Research Institute, Cheongju, Republic of Korea; 4 State Key Laboratory of Advanced Medical Materials and Devices, Tianjin Key Laboratory of Biomedical Materials, Institute of Biomedical Engineering, Chinese Academy of Medical Sciences and Peking Union Medical College, Tianjin, China

**Keywords:** antibacterial, antitumor, photodynamic therapy (PDT), photosensitive agents, reactive oxygen species

## Introduction

Photodynamic therapy (PDT) exemplifies the convergence of photochemistry, molecular biology, and clinical oncology, transitioning from a niche dermatological modality into a sophisticated multidomain platform with applications spanning oncology, antimicrobial therapy, inflammatory diseases, and regenerative medicine. Recent advances in photosensitizer chemistry, nanotechnology integration, and immunological understanding position PDT as a cornerstone precision medicine strategy, distinct from conventional therapy through its inherent spatiotemporal control and capability to induce immunogenic cell death.

## PDT mechanisms: beyond phototoxicity to immunotherapy

PDT operates through a coordinated triad, such as photosensitizer (PS), light of a specific wavelength, and molecular oxygen, which collectively generate reactive oxygen species (ROS), predominantly singlet oxygen and hydroxyl radicals. These highly reactive species exert cytotoxic effects by irreversibly oxidizing lipids, proteins, and nucleic acids. ROS-induced damage activates multiple regulated cell death pathways, including intrinsic and extrinsic apoptosis, autophagy, and ferroptosis, providing mechanistic redundancy that may limit resistance development. In addition to direct tumor cell killing, increasing evidence highlights PDT’s ability to provoke immunogenic cell death (ICD), characterized by the release of damage-associated molecular patterns (DAMPs) that stimulate dendritic cell maturation and promote effector T-cell recruitment. This immunological facet repositions PDT from a purely localized cytotoxic intervention to a systemic immune-activating strategy.

Recent mechanistic studies demonstrate that PDT-induced immunogenic cell death (ICD) is contingent upon mitochondrial dysfunction, accompanied by cytochrome c release and activation of the NLRP3 inflammasome, thereby engaging both innate and adaptive immune responses. PDT further reshapes the tumor microenvironment (TME) through several coordinated mechanisms, including direct endothelial damage and vascular shutdown, polarization of macrophages toward pro-inflammatory M1 phenotypes, enhanced recruitment and activation of cytotoxic T lymphocytes, and attenuation of immunosuppressive regulatory T cell (Treg) populations. These immunomodulatory effects are amplified when PDT is combined with immune checkpoint inhibitors, establishing a paradigm of synergistic immune activation.

This topic provides an overview of PDT, outlining the mechanisms of PDT-induced immune activation and the applications of PSs in this field (Cai et al.). We also examine the recent progress and future direction of photodynamic nanodrugs, which significantly improve therapeutic outcomes by reducing systemic toxicity through enhanced solubility, biodistribution and accumulation of photosensitizers (Ma et al.). If you are interested in light-responsive hydrogels, the review by Fan et al. covers those based on natural products, their use in biomedicine, and their preparation methods. For the treatment of skin cancers, we summarized how photodynamic therapy works, its uses, benefits and drawbacks, and future advancements (Hua et al.).

## Evolution of photosensitizers: third-generation precision agents

PDT has advanced through three generations of photosensitizers. First-generation agents exhibit poor selectivity and prolonged cutaneous photosensitivity. Second-generation compounds demonstrated improved selectivity and therapeutic efficacy, yet tissue penetration remained restricted to ∼5–10 mm due to optical absorption within the visible spectrum. Third-generation photosensitizers, currently emerging as research frontiers, integrate nanotechnology, target ligands, and stimulus-responsive systems to achieve enhanced selectivity and greater depth of action.

Recent advances include near-infrared (NIR) photosensitizers activatable at 700–900 nm wavelengths, enabling increased tissue penetration and reduced autofluorescence. Nanoformulation strategies, such as polymeric nanocarriers, lipid-based systems, and upconversion nanoparticles (UCNPs), improve photosensitizer solubility, bioavailability, and cellular internalization while minimizing systemic toxicity. Activatable photosensitizers respond selectively to tumor microenvironment characteristics (acidic pH, hypoxia, specific enzymes), restricting PS activation exclusively within diseased tissue. Organelle-targeted PSs localize ROS generation to mitochondria or lysosomes, enhancing apoptotic signaling. Additionally, metal-based photosensitizers, porphyrin-metal complexes, gold nanoparticle conjugates, and photocatalytic materials introduce synergistic ROS generation pathways and photothermal effects.

This topic report a novel photosensitizer. Rong et al. investigated the therapeutic effects and mechanisms of LD4-PDT on ulcerative colitis. Zhu et al. reported on the therapeutic potential of the photosensitizer LD4 in a murine model of pulmonary fibrosis and demonstrate its anti-fibrotic effects in bleomycin-induced pulmonary fibrosis. Interestingly, this topic included two papers: one on enhancing photodynamic therapy and the other on ameliorating skin photoaging (Macri et al.; Zhou et al.).

## Clinical oncology applications: from dermatology to systemic disease

PDT has its most established clinical applications in non-melanoma skin cancers (NMSC) (Chen et al.). Actinic keratoses, basal cell carcinomas, and squamous cell carcinomas respond effectively to topical PDT using 5-aminolevulinic acid (ALA) or methylaminolevulinate (MAL) photosensitizers. Recent bibliometric analyses of NMSC-PDT literature indicate a predominant research emphasis on immune mechanisms: T cell polarization, dendritic cell maturation, and Th17-driven inflammation emerge as key effector pathways. Emerging daylight PDT modalities, employing natural sunlight instead of clinic-based light sources, aim to broaden treatment accessibility and minimize procedural discomfort, addressing a significant barrier to wider clinical implementation.

Systemic oncology applications remain largely preclinical but demonstrate considerable promise. Breast cancer, particularly triple-negative breast cancer (TNBC), represents a strategic target for PDT-immunotherapy combinations (Liu et al.) TNBC displays intrinsic immunogenicity yet paradoxically sustains an immunosuppressive TME, rendering checkpoint blockade alone insufficiently effective. Recent studies show that combining PDT with anti-PD-L1 antibodies achieves sustained tumor control and inhibits metastasis in murine TNBC models through coordinated enhancement of cytotoxic T cell infiltration and reduction of Treg populations. Clinical trials (NCT04305795) are currently evaluating cetuximab-targeted IR700 PDT combined with PD-1 inhibitors (pembrolizumab) in head and neck squamous cell carcinoma, representing a pivotal inflection point in PDT clinical translation.

Mechanistically, PDT disrupts tumor vasculature and enhances intratumoral vascular permeability, thereby facilitating the delivery of large therapeutic antibodies. Furthermore, PDT-induced vascular shutdown generates transient hypoxia that paradoxically primes adaptive immune responses via hypoxia-inducible factor (HIF)-A signaling. Integration of PDT with conventional chemotherapy and targeted radiotherapy is under active investigation, with early-phase data suggesting additive or synergistic cytotoxic effects while potentially reducing required drug dosages.

## Antimicrobial photodynamic therapy: addressing antibiotic resistance

Antimicrobial photodynamic therapy (aPDT) addresses a critical global health challenge: antimicrobial resistance (AMR) is responsible for >1.27 million deaths annually and is projected to surpass cancer mortality by 2050 without effective intervention. aPDT provides mechanistic advantages not achievable with conventional antibiotics. ROS generation directly disrupts bacterial membranes, nucleic acids, and essential enzymes targets fundamentally inaccessible to classical resistance mechanisms. Importantly, aPDT-resistant bacterial strains have not been observed even after >25 repeated photoinactivation cycles under laboratory conditions, suggesting that resistance development is highly unlikely.

Current aPDT applications effectively target both gram-positive and gram-negative pathogens, including methicillin-resistant *Staphylococcus aureus* (MRSA), *Pseudomonas aeruginosa*, and extended-spectrum beta-lactamase (ESBL)-producing *Escherichia coli.* Emerging photosensitizers such as methylene blue, toluidine blue, and novel agents like LD4 demonstrate improved antimicrobial efficacy against these organisms. The combinatorial use of aPDT with conventional antibiotics shows particular promise, as PDT disrupts bacterial biofilm architecture and impairs efflux pump activity, rendering residual bacteria highly susceptible to antibiotic penetration and action. aPDT antibiotic combinations exhibit significantly reduced minimum inhibitory concentrations (MICs) against multidrug-resistant pathogens.

Intracellular pathogens, including *Mycobacterium tuberculosis* and *Mycobacterium fortuitum*, pose distinct challenges; their lipid-rich cell walls and slow growth kinetics confer intrinsic resistance to aPDT. However, emerging strategies integrating aPDT with immunomodulatory agents or permeability enhancers demonstrate early promise in drug-resistant and extensively drug-resistant tuberculosis models. Osteomyelitis, a notoriously difficult-to-treat bone infection, represents a compelling aPDT indication due to its localized pathology and substantial antibiotic penetration limitations; combined 5-aminolevulinic acid-mediated PDT with conventional antibiotics shows enhanced therapeutic outcomes in preclinical osteomyelitis models.

In our topic, Wang et al. also reports antibacterial photodynamic therapy using lysine-porphyrin conjugate LD4 is effective against methicillin-resistant *S. aureus*, *P. aeruginosa*, and *E. coli*, but is ineffective against *Candida albicans* and *M. tuberculosis*. Another study looked at how well 5-aminolevulinic acid-mediated photodynamic therapy works in treating tibial osteomyelitis in rabbits (Zuo et al.).

## Expanding therapeutic frontiers: Inflammatory and fibrotic diseases

Emerging evidence reveals PDT’s therapeutic potential in non-malignant conditions characterized by dysregulated inflammatory and fibrotic remodeling. Rong et al. Ulcerative colitis, a chronic inflammatory bowel disease, responds to aPDT through multiple mechanisms: direct epithelial ROS-mediated injury eliminates pathogenic microbiota; inhibition of the NF-κB pathway reduces pro-inflammatory cytokine (IL-6, TNF-α) production; and colonic epithelial barrier integrity is restored via enhanced antioxidant enzyme expression (superoxide dismutase, catalase). aPDT using the novel photosensitizer LD4 induces disease remission in dextran-sodium sulfate (DSS)-induced colitis models and reestablishes dysbiotic microbiota composition toward eubiotic states.

Pulmonary fibrosis a progressive, fibrosis-dominated disorder with poor prognosis, represents an unconventional yet promising aPDT target (Zhu et al.). Bleomycin-induced pulmonary fibrosis models treated with LD4-PDT show marked attenuation through modulation of macrophage phenotype: PDT inhibits M2 macrophage differentiation and decreases mannose receptor 1 (MRC1) expression, thereby suppressing the profibrotic secretory program (transforming growth factor-beta, TGF-β; matrix metalloproteinases, MMPs). This mechanistic insight, that PDT-driven metabolic reprogramming shifts macrophage immunometabolism from oxidative phosphorylation (OXPHOS) toward glycolysis, thereby limiting fibrotic phenotypes, opens entirely new therapeutic avenues for PDT.

Beyond infection and malignancy, PDT exhibits emerging cosmeceutical applications. Vicenin-2, a natural compound with PDT-like immunomodulatory effects, mitigates photoaging through modulation of macrophage polarization and N6-methyladenosine (m6A)-mediated epigenetic reprogramming. These findings highlight PDT’s capacity to reprogram tissue immune surveillance and metabolic state mechanisms distinct from direct cytotoxicity.

## Translational barriers and future perspectives

Despite substantial scientific advances, translation of PDT from bench to bedside continues to face significant challenges. First, photosensitizer selectivity remains suboptimal; systemic administration leads to off-target accumulation and nonspecific phototoxicity, particularly problematic in the context of cutaneous photosensitivity. Next-generation strategies include active targeting via monoclonal antibody conjugates, peptide ligands, and aptamers enabling PS accumulation >100-fold within tumors, activatable photosensitizers responsive specifically to tumor-associated proteases or acidic microenvironmental pH, and *ex vivo* CAR-T cell engineering incorporating PS, enabling light-triggered cytotoxic T lymphocyte activation.

Second, tissue penetration depth, intrinsically limited to ∼10 mm for visible wavelengths, necessitates interstitial or intravascular light delivery for deep-seated tumors. NIR photosensitizers (700–900 nm) extend penetration to ∼20 mm, whereas photoacoustic imaging, X-ray-activated scintillating nanoparticles, and upconversion nanotechnology provide emerging strategies to overcome optical attenuation constraints.

Third, tumor hypoxia, which limits ROS generation, requires combination with oxygen-delivery nanosystems or hypoxia-alleviating immunotherapies (anti-VEGF agents, anti-angiogenic kinase inhibitors).

Fourth, standardization of PDT protocols remains inconsistent across preclinical and clinical studies. Variability in reporting of light dosimetry (fluence, fluence rate, wavelength), photosensitizer dose, drug–light intervals, and outcome measures limits meta-analysis and evidence integration. Consensus guidelines, prospective clinical registries, and harmonized protocols across institutions are essential.

Finally, a comprehensive mechanistic understanding of PDT-induced immune responses in patients remains incomplete; most clinical evidence arises from small, single-arm trials. Robust randomized controlled trials incorporating mechanistic immunological endpoints (TCR sequencing, flow cytometry, and comprehensive immune profiling) are required to validate preclinical findings.

## Conclusion and vision for precision photomedicine

This Research Topic highlights the evolution of PDT from an empirical dermatological intervention into a rationally engineered, mechanism-driven therapeutic platform with applications spanning oncology, antimicrobial therapy, and inflammation. The convergence of three technological advances, photosensitizer chemistry, nanotechnology integration, and immunobiological insights, generates unprecedented therapeutic opportunities. As photosensitizer selectivity improves through targeting ligands, tissue penetration is extended via NIR and upconversion strategies, and biomarkers enabling patient stratification are validated, PDT’s clinical impact will expand considerably. Future directions emphasize rational design of PDT agents incorporating immunogenic features, integration with checkpoint blockade, chemotherapy, and radiotherapy for synergistic efficacy, real-time dosimetry and tissue oxygenation monitoring, clinical evaluation in resistant malignancies and refractory infections, and extension into inflammatory and fibrotic diseases through mechanistic insight.

